# Caffeic Acid *O*-Methyltransferase Gene Family in Mango (*Mangifera indica* L.) with Transcriptional Analysis under Biotic and Abiotic Stresses and the Role of *MiCOMT1* in Salt Tolerance

**DOI:** 10.3390/ijms25052639

**Published:** 2024-02-24

**Authors:** Huiliang Wang, Zhuoli Chen, Ruixiong Luo, Chen Lei, Mengting Zhang, Aiping Gao, Jinji Pu, He Zhang

**Affiliations:** 1 Key Laboratory of Green Prevention and Control of Tropical Plant Diseases and Pests (Hainan University), Ministry of Education, Key Laboratory of Biotechnology of Salt Tolerant Crops of Hainan Province, Hainan University, Haikou 570228, China; 21220951320091@hainanu.edu.cn (H.W.); 21220951310147@hainanu.edu.cn (Z.C.); 21220951320114@hainanu.edu.cn (M.Z.); 2 National Key Laboratory for Tropica1 Crop Breeding, Key Laboratory of Integrated Pest Management on Tropical Crops, Ministry of Agriculture and Rural Affairs, Chinese Academy of Tropical Agricultural Sciences Environment and Plant Protection Institute, Haikou 571101, China; clei1220@163.com (C.L.); cataspjj@163.com (J.P.); 3 Chinese Academy of Tropical Agricultural Sciences Tropical Crops Genetic Resources Institute, National Key Laboratory for Tropical Crop Breeding, Laboratory of Crop Gene Resources and Germplasm Enhancement in Southern China, Ministry of Agriculture and Rural Affairs, Key Laboratory of Tropical Crops Germplasm Resources Genetic Improvement and Innovation of Hainan Province, Haikou 571101, China; luoruixiong@163.com

**Keywords:** *COMT* gene family, salt tolerance, transcriptional analysis, transient overexpression

## Abstract

Caffeic acid *O*-methyltransferase (*COMT*) participates in various physiological activities in plants, such as positive responses to abiotic stresses and the signal transduction of phytohormones. In this study, 18 *COMT* genes were identified in the chromosome-level reference genome of mango, named *MiCOMTs*. A phylogenetic tree containing nine groups (I-IX) was constructed based on the amino acid sequences of the 71 COMT proteins from seven species. The phylogenetic tree indicated that the members of the MiCOMTs could be divided into four groups. Quantitative real-time PCR showed that all *MiCOMT* genes have particularly high expression levels during flowering. The expression levels of *MiCOMTs* were different under abiotic and biotic stresses, including salt and stimulated drought stresses, ABA and SA treatment, as well as *Xanthomonas campestris* pv. *mangiferaeindicae* and *Colletotrichum gloeosporioides* infection, respectively. Among them, the expression level of *MiCOMT1* was significantly up-regulated at 6–72 h after salt and stimulated drought stresses. The results of gene function analysis via the transient overexpression of the *MiCOMT1* gene in *Nicotiana benthamiana* showed that the *MiCOMT1* gene can promote the accumulation of ABA and MeJA, and improve the salt tolerance of mango. These results are beneficial to future researchers aiming to understand the biological functions and molecular mechanisms of *MiCOMT* genes.

## 1. Introduction

Caffeic acid *O*-methyltransferase (*COMT*) is a multifunctional enzyme that catalyzes *O*-methylation with various substrates [1,2]. Previous studies have shown that the *COMT* gene family is generally composed of multiple members, such as rapeseed (*Brassica napus*) (25) [3], catalpa bungei (*Catalpa bungei*) (23) [4], birch (*Betula pendula*) (25) [5], blueberry (*Vaccinium corymbosum*) (92) [6], soybean *(Glycine max*) (55) [7], and rice (*Oryza sativa*) (33) [8]. *COMT* plays a regulatory role in various stress responses in plants, such as salt [9], cold [7], drought [10], and the signal transduction of phytohormones. Bugos and colleagues were the first to clone the *COMT* gene from poplar (*Populus*) [11]. At present, the *COMT* genes of many plants have been studied, such as Arabidopsis (*Arabidopsis thaliana*) [12] and barley (*Hordeum vulgare*) [13]. In rice roots, *COMT* gene expression was down-regulated under salt treatment [8]. Under salt stress, *CrCOMT* (*Carex rigescens*-*COMT*) transgenic Arabidopsis has higher salt-responsive gene transcriptional activity than wild-type samples [14]. *TaCOMT* (*Triticum aestivum*-*COMT*) transgenic Arabidopsis seedlings were treated with different concentrations of mannitol and PEG6000, and their total root length, number of lateral roots, and fresh weight were significantly greater than those of the wild-type sample [15]. The overexpression of *SlCOMT* in tomato (*Solanum lycopersicum*) could inhibit the accumulation of H_2_O_2_ and improve salt tolerance [16]. It can be seen that *COMT* consistently improves the salt tolerance of plants, which is mainly due to the significantly increased osmotic regulatory and antioxidant capacity in these transgenic plants under salt stress [17]. However, there is currently no report on the identification and functional analysis of mango *COMT* genes.

Mango (*Mangifera indica* L., 2n = 2x = 40) is one of the most important economic tropical fruits in the world [18], and it is grown in more than 100 countries and regions. Mango fruit is highly appreciated for its delicious taste, exotic flavor, and nutritional value [19]. Fruits are important sources of carbohydrates, proteins, fats, energy, vitamins, micronutrients, dietary fiber, phenolic compounds, and other phytochemicals [20]. Mango is mainly cultivated in salinized hilly, mountainous, and coastal areas. Soil salinization is a key obstacle to sustainable mango production [21]. The main symptoms are varying degrees of leaf tip or leaf edge wilt, premature fall of branches and leaves, fewer flowers and fruits, and even the death of the whole plant [22]. In recent years, due to the changes in climate and human factors, the situation regarding global soil salinization has been deteriorating, and more than 800 million hectares of land have been affected by soil salinization [23]. Soil salinization is also one of the main abiotic stress factors in global agricultural production [24]. High concentrations of salt ions in soil will reduce the water potential of soil [25]. Excessive salt ions passively transported into plant cells through ion channels and transpiration will directly damage plant leaf cells [26,27], inhibit nutrient absorption and photosynthesis [28], reduce plant water absorption capacity, and hinder plant growth [29]. In rice, salt stress may stunt growth [30]. In apple (*Malus domestica*) growing areas, high concentrations of salt ions in soil reduce fruit yield and quality [31]. This is mainly caused by the toxicity of ions and the accumulation of reactive oxygen species (ROS) during salt stress [32], including hydrogen peroxide (H_2_O_2_), superoxide anion (O^2−^), and malondialdehyde (MDA) [33]. Thus, the physiological and biochemical indices of plants may be altered. For example, the contents of water and chlorophyll are decreased, but the content of MDA is increased following salt stress in JUNCAOs (*Pennisetum giganteum*) [34]. In hyssop (*Hyssopus officinalis*) plants, phenols and anthocyanins are significantly increased upon salt stress treatment [35]. SA- and MeJA-pretreated sorghum (*Sorghum bicolor*) plants show substantial decreases in ROS accumulation under salt stress [36,37]. Excess salt leads to an increase in the content of ABA in rice [38]. Therefore, understanding the mechanisms of plant tolerance to salt stress is essential to the growth and development of plants in salinized soils.

In this study, we aimed to explore the molecular characteristics and phylogenetic tree of the *COMT* gene family in mango. Their differential expression patterns were analyzed in different mango tissues and under different stress treatments. In particular, the function of the *MiCOMT1* gene in regulating salt stress tolerance was revealed. The outcomes of this study will provide a fundamental basis for the functional characterization of *MiCOMT* gene members, especially regarding salt tolerance.

## 2. Results

### 2.1. Identification and Phylogenetic Tree Analysis of MiCOMT Gene Family

**Figure 1:**
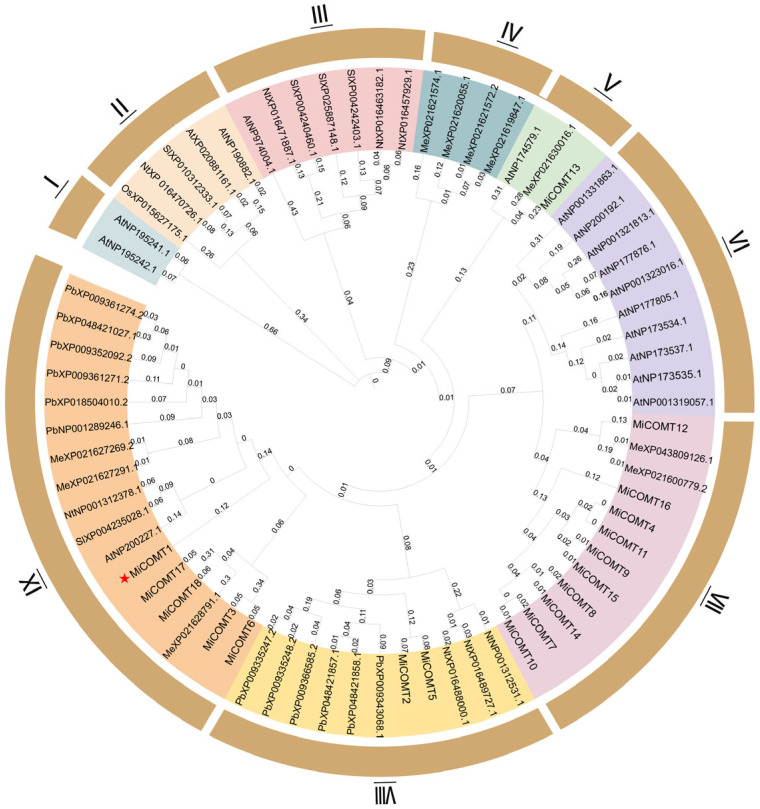
Phylogenetic relationship of COMTs in different species using complete protein sequences. The neighbor-joining (NJ) phylogenetic tree was constructed with the Poisson model with 1000 bootstrap replicates using MEGA 11 software. The tree was generated from an amino acid sequence alignment of *M. indica* (*Mi*) (18 *MiCOMTs*), *A. thaliana* (*At*) (17 members), *M. esculenta* (*Me*) (10 members), *N. tabacum* (*Nt*) (8 members), *S. lycopersicum* (*Sl*) (5 members), *O. sativa* (*Os*) (1 member), and *P. bretschneideri* (*Pb*) (12 members). The phylogenetic tree was categorized into nine groups, highlighted using different colors. The numerical value on the branch indicates the genetic distance. I–IX indicates grouping.Red star means emphasized that the later *MiCOMT1* is the main body of research in this article.

To understand the evolutionary expansion of the mango *COMT* gene family, a phylogenetic tree was constructed using MEGA 11 software, based on multiple alignments. We attempted to characterize the phylogenetic relationship among 71 COMT (Table S1) proteins of *M. indica* (*Mi*) (18 *MiCOMTs*), *A. thaliana* (*At*) (17 members), *Manihot esculenta* (*Me*) (10 members), *Nicotiana tabacum* (*Nt*) (8 members), *S. lycopersicum* (*Sl*) (5 members), *O. sativa* (*Os*) (1 member), and *Pyrus bretschneideri* (*Pb*) (12 members), as shown in Figure 1 and Figure S1. According to the phylogenetic tree, 71 COMT proteins could be categorized into nine groups (I-IX). MiCOMT4, MiCOMT7, MiCOMT8, MiCOMT9, MiCOMT10, MiCOMT11, MiCOMT12, MiCOMT14, MiCOMT15, and MiCOMT16 were distributed in group VII, which is equivalent to 55.56% of the total number of MiCOMT proteins. MiCOMT2 and MiCOMT5 were distributed in group VIII. MiCOMT1, MiCOMT3, MiCOMT6, MiCOMT17, and MiCOMT18 were distributed in group IX. Only MiCOMT13 was distributed in group V, which is equivalent to 5.56% of the total number of MiCOMT proteins. From the phylogenetic tree (Figure 1), it can be seen that there were seven pairs of direct homologous genes of MiCOMT proteins, and one pair of direct homologous genes in cassava and mango were identified.

### 2.2. Structural and Motif Analysis of MiCOMT Proteins

**Figure 2:**
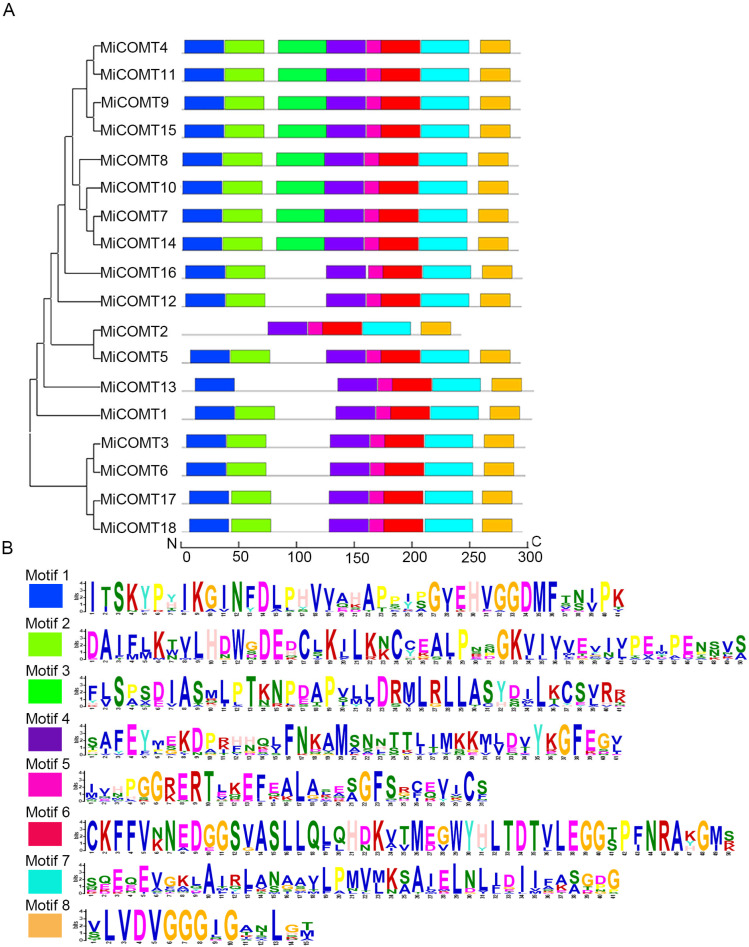
The structural and motif analysis of the MiCOMTs: (**A**) The phylogenetic tree was constructed with MEGA 11 software using protein sequences of the 18 MiCOMT proteins. The motifs were characterized using the MEME website and TBtools v1.098768 software with the number of motifs set to 8, and the motif discovery mode was classic mode. The eight motifs are named Motif 1 to Motif 8, and are represented with different color boxes. (**B**) The consensus sequence of 8 motifs.

To explore the structural characteristics and motif conservation of COMT proteins, phylogenetic analysis and MEME were used. We further investigated the structural diversity of MiCOMT proteins. The results of the motif analysis showed that all MiCOMTs contained Motif 4, Motif 5, Motif 6, Motif 7, and Motif 8 (Figure 2A,B), forming the conserved domain of the COMT protein. Thus, it was proved that 18 MiCOMT proteins belonged to the COMT protein family. MiCOMT2 failed to exhibit Motif 1, Motif 2, and Motif 3, while Motif 2 was not detected in MiCOMT13. More than 88.89% of MiCOMT proteins contained seven motifs.

### 2.3. Chromosomal Distribution and Syntenic Analysis of MiCOMT Genes

**Figure 3:**
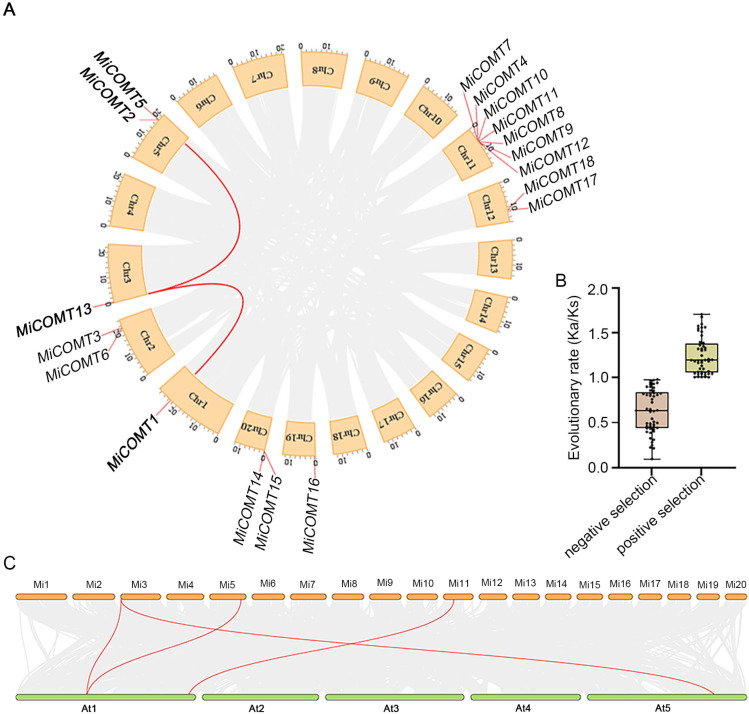
The collinearity and Ka/Ks of COMT proteins: (**A**) Collinearity analysis of the *COMT* gene family in mango. Each colored square on the edge of the circle represents mango chromosomes. The gray lines indicate collinear pairs of all mango genes, and the red lines represent collinear pairs of *MiCOMTs*. (**B**) Distribution of the Ka/Ks values of *MiCOMTs*. (**C**) Collinearity analysis of *COMT* genes among mango and Arabidopsis. The gray lines represent the collinear blocks in the genomes of mango and Arabidopsis, and the red line represents the collinear *COMT* gene pairs.

To analyze the positions of the *MiCOMT* genes in the mango genome, chromosomal mapping was performed, and gene duplication events were analyzed. The results suggest that 18 *MiCOMT* genes were heterogeneously distributed on 8 of the 20 chromosomes. Among these genes, the figure shows seven genes in chr 11; two genes in chrs. 2, 5, 12, and 20; and one gene in chrs. 1, 3, and 19, which correspond to the 18 genes identified. The Ka value, Ks value, and Ka/Ks ratios for the paralog *MiCOMT* gene pairs were calculated using Tbtools. The *MiCOMT* genes had 98 duplicate pairs (Table S2), of which 50 (51.0%) had Ka/Ks values less than 1, and the remaining 48 had Ka/Ks values between 1.0 and 1.8, indicating that *MiCOMT* genes underwent neutral selection during the rapid evolution stage (Figure 3B). To explore the phylogenetic mechanism of the *MiCOMTs*, a comparison of interspecific synteny among mango and Arabidopsis was conducted. A direct comparison of the homologous gene pairs for all chromosomes showed that three *MiCOMT* genes were synonymous with Arabidopsis. It can be observed that the *COMT* gene of mango had a high sequence similarity and close homologous evolutionary relationship with Arabidopsis (Figure 3C).

### 2.4. Analysis of the Tissue Expression Patterns of MiCOMT Genes

**Figure 4:**
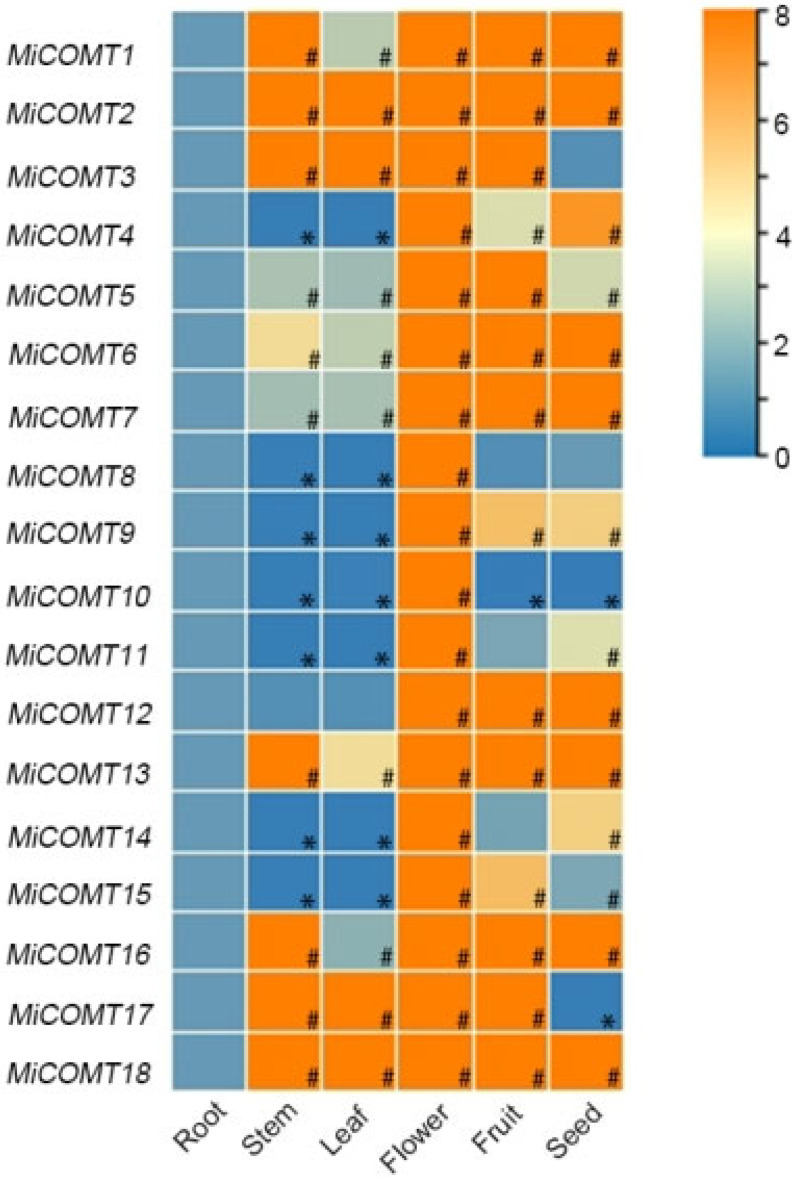
The expression of *MiCOMT* genes in different mango tissues. The colors spanning from blue to orange represent increasing levels of gene expression. The level of expression in root was used as a reference to determine up- or down-regulation in the other plant tissues. The horizontal axis represents the different tissue parts of the mango, and the vertical axis represents the *MiCOMT* genes. “#” represents significantly up-regulated gene expression. “*” represents significantly down-regulated gene expression.

The expression profiles of 18 *MiCOMT* genes in six tissues (root, stem, leaf, flower, fruit, and seed) were estimated using qRT-PCR, with the root as the control group (Figure 4 and Table S3). All *MiCOMT* genes members were significantly up-regulated in flower, and *MiCOMT1*, *MiCOMT2*, *MiCOMT5*, *MiCOMT6*, *MiCOMT7*, *MiCOMT13*, *MiCOMT16*, and *MiCOMT18* were significantly up-regulated in all tissues. However, the relative expressions of *MiCOMT4*, *MiCOMT8*, *MiCOMT9*, *MiCOMT10*, *MiCOMT11*, *MiCOMT14*, and *MiCOMT15* in leaf and stem were significantly down-regulated. Comprehensive analysis showed that the different members of *MiCOMTs* had specific expression patterns in different tissues, and 18 *MiCOMT* members had high transcription levels in flower.

### 2.5. Differential Expression Analysis of MiCOMT Genes under Salt Stress and Drought Stress

**Figure 5:**
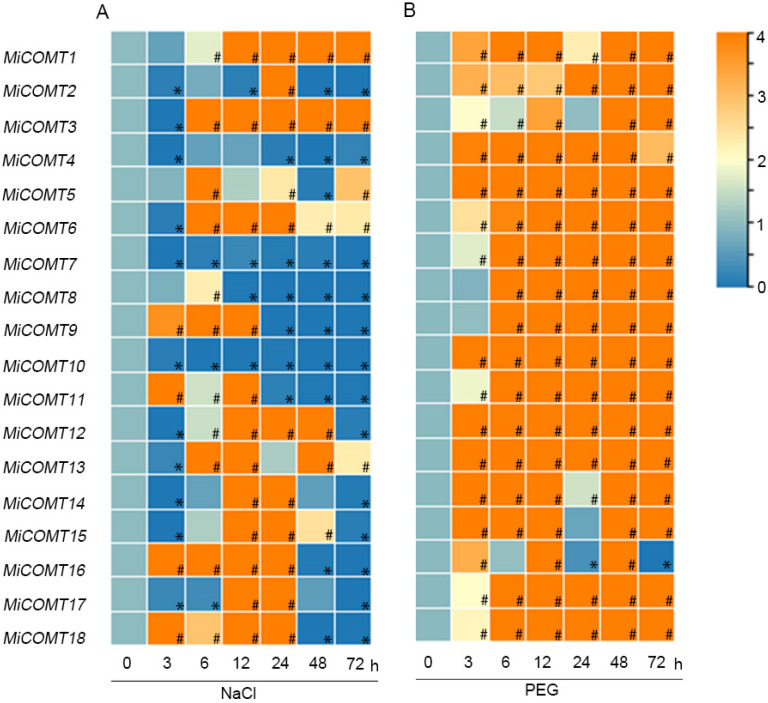
Expression patterns of *MiCOMT* genes under salt and drought stresses. Mango seedlings were treated with 300 mmol·L^−1^ NaCl (**A**) and 30% PEG6000 (**B**) for 0, 3, 6, 12, 24, 48, and 72 h. The level of expression at 0 h was used as a reference to determine up- or down-regulation at other time points. The colors spanning from blue to orange represent increasing levels of gene expression. “#” represents significantly up-regulated gene expression. “*” represents significantly down-regulated gene expression.

We analyzed the expression patterns of *MiCOMT* genes under salt stress and simulated drought stress using qRT-PCR. Under salt stress (Figure 5A and Table S4), *MiCOMT7* and *MiCOMT10* were significantly down-regulated at all time points in mango leaves, while *MiCOMT1* and *MiCOMT3* were significantly up-regulated between 6 and 72 h. Under drought stress (Figure 5B and Table S5), all *MiCOMT* genes were significantly up-regulated at 12 and 48 h in mango leaves. The expression of 13 members (72.2%) increased significantly at all time points. These results showed that the expression patterns of *MiCOMT* genes were different under salt and drought stresses, but *MiCOMT1* had the potential to exhibit an up-regulation response to both types of stress.

### 2.6. Differential Expression Analysis of MiCOMT Genes in Response to ABA and SA Treatments

**Figure 6:**
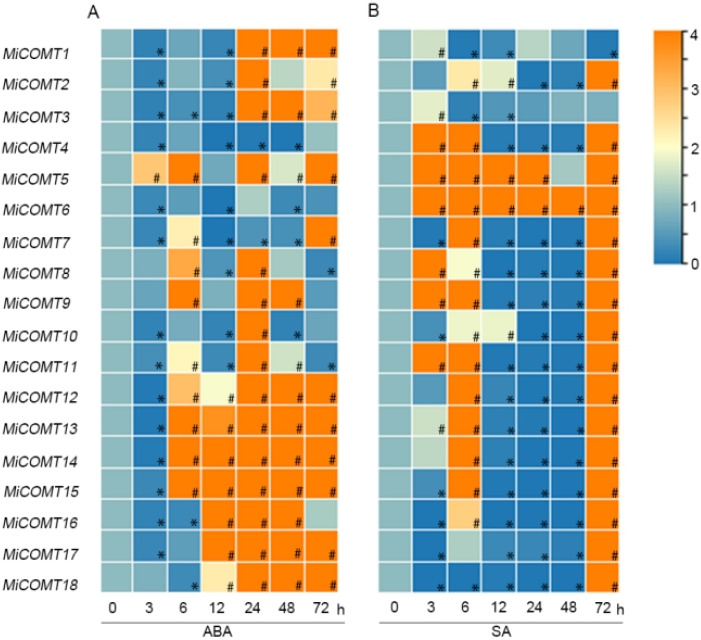
Expression patterns of *MiCOMT* genes under ABA and SA treatment. Mango seedling leaves were treated with 5 mmol·L^−1^ ABA (**A**) and 5 mmol·L^−1^ SA (**B**) for 0, 3, 6, 12, 24, 48, and 72 h. The level of expression at 0 h was used as a reference to determine up- or down-regulation at other time points. The colors spanning from blue to orange represent increasing levels of gene expression. “#” represents significantly up-regulated gene expression. “*” represents significantly down-regulated gene expression.

ABA and SA activated the *MiCOMT* genes’ transcription levels. During ABA treatment (Figure 6A and Table S6), *MiCOMT12*, -*13*, -*14*, and -*15* were significantly up-regulated between 6 and 72 h in mango leaves. A total of 15 *MiCOMT* gene members showed significant increases in transcription levels at 24 h in mango leaves. More than 66% of *MiCOMT* genes were up-regulated at 24-72 h in mango leaves. During SA treatment (Figure 6B and Table S7), *MiCOMT6* was significantly up-regulated at all time points in mango leaves. A total of 16 *MiCOMT* gene members were significantly up-regulated at 72 h in mango leaves, while *MiCOMT1* was significantly down-regulated at 6, 12, and 72 h. Twelve *MiCOMT* genes were significantly down-regulated at 12-48 h in mango leaves. These results suggest that the *MiCOMT* gene responds to ABA and SA treatments.

### 2.7. Differential Expression Analysis of MiCOMT Gene Responses to Pathogen Infection

**Figure 7:**
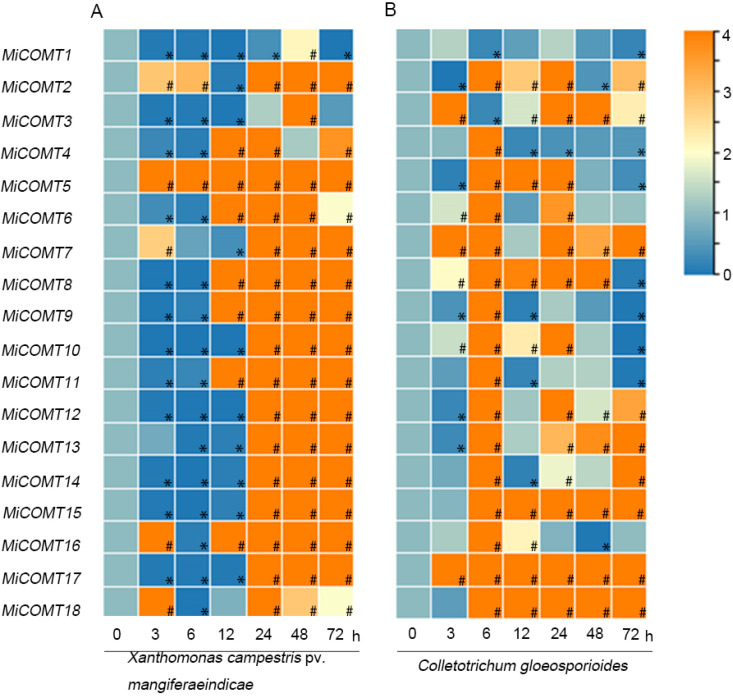
Expression patterns of *MiCOMT* genes during infection with *X. campestris* pv. *mangiferaeindicae* and *C. gloeosporioides*. Mango seedling leaves were treated with 2 × 10^6^ cfu·mL^−1^ of *X. campestris* pv. *mangiferaeindicae* suspension (**A**) and 2 × 10^7^ conidia·mL^−1^ suspension of *C. gloeosporioides* (**B**) for 0, 3, 6, 12, 24, 48, and 72 h. The level of expression at 0 h was used as a reference to determine up- or down-regulation at other time points. The colors spanning from blue to orange represent increasing levels of gene expression. “#” represents significantly up-regulated gene expression. “*” represents significantly down-regulated gene expression.

To investigate the possible role of *MiCOMTs* in plant-pathogen interactions, qRT–PCR was used to analyze the response of mango leaves infected with *X. campestris* pv. *mangiferaeindicae* and *C. gloeosporioides* in comparison to 0 h. During infection with *X. campestris* pv. *mangiferaeindicae* (Figure 7A and Table S8), *MiCOMT5* was significantly up-regulated at all time points in mango leaves. At 24-72 h, the transcription levels of 15 *MiCOMT* gene members were significantly increased in mango leaves, and the *MiCOMT1* and *MiCOMT3* genes were significantly down-regulated at 0-12 h. During infection with *C. gloeosporioides* (Figure 7B and Table S9), the transcriptional levels of *MiCOMT17* were consistently up-regulated at all time points in mango leaves. *MiCOMT1* and *MiCOMT3* were significantly down-regulated at 6 h in mango leaves, while 16 members were significantly up-regulated at 6 h in mango leaves. These results showed that *MiCOMT* genes had different expression patterns in response to both pathogenic bacteria and fungi.

### 2.8. Transient Overexpression of MiCOMT1 in N. benthamiana Increased Salt Tolerance

**Figure 8:**
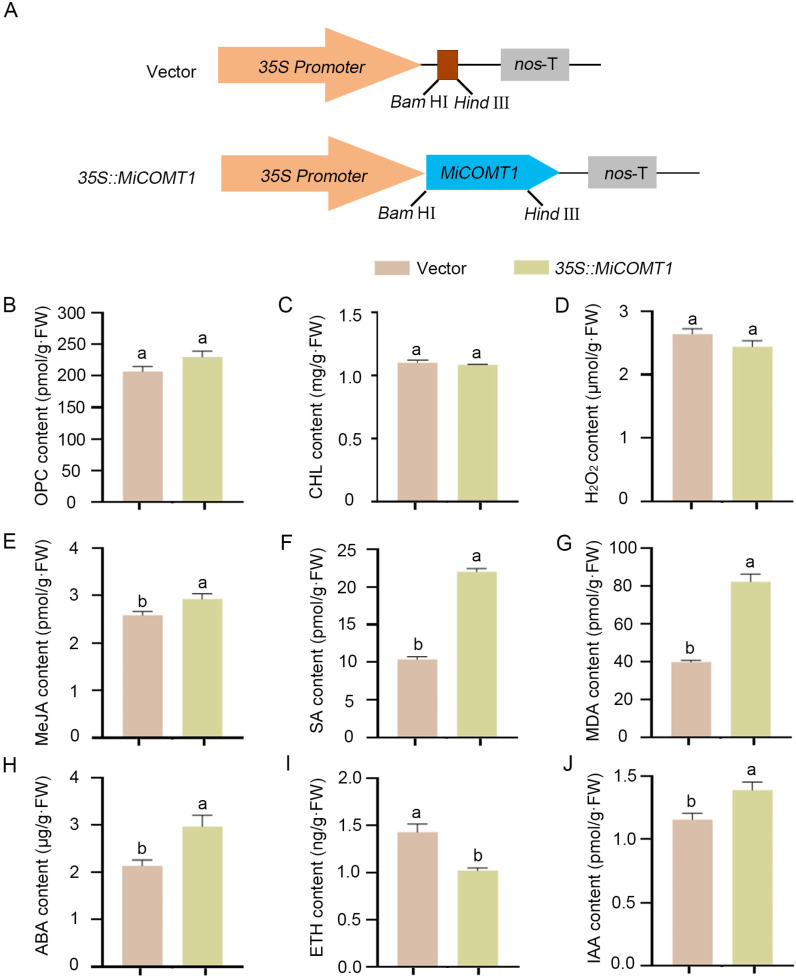
At 3 dpi, gene-overexpressing *N. benthamiana* leaves were used for physiological and biochemical analysis. (**A**) Schematic diagram of the control (vector) and *35S::MiCOMT1*. (**B**–**J**) Each experiment had three replicates and six *N. benthamiana* seedlings per replicate. Oligomeric Proanthocyanidin (OPC), chlorophyll (CHL), hydrogen peroxide (H_2_O_2_), methyl jasmonic acid (MeJA), salicylic acid (SA), malondialdehyde (MDA), abscisic acid (ABA), ethylene (ETH), and auxin (IAA) contents of *N. benthamiana* seedlings were treated as described. All data represent mean ± SE for three biological replications. Statistical significance was determined using Duncan’s test, and “a and b” represent significant differences.

In vivo, to further reveal the role of the *MiCOMT1* gene in salt tolerance, the phenotype was analyzed via the transient overexpression of *MiCOMT1* in *N. benthamiana* leaves. For that, the recombinant plasmid *35S::MiCOMT1* and the empty vector were introduced into *N. benthamiana* using the heat shock method (Figure 8A). *N. benthamiana* leaves were infected with *Agrobacterium* containing *35S::MiCOMT1* or control for 3 days. *MiCOMT1*-gene-overexpressing *N. benthamiana* leaves were used for physiological assays, including Oligomeric Proanthocyanidin (OPC), chlorophyll (CHL), hydrogen peroxide (H_2_O_2_), methyl jasmonic acid (MeJA), salicylic acid (SA), malondialdehyde (MDA), abscisic acid (ABA), ethylene (ETH), and auxin (IAA) contents (Figure 8B–J). Using the control group as the x-axis and the *35S::MiCOMT1* group as the y-axis, simple linear regression analysis was performed using GraphPad Prism 9 software, and the results showed a significant positive correlation (y = 0.9559x + 10.05, R^2^ = 0.9619, *p* < 0.0001; Figure S2). These results showed that compared with the control group, the contents of anthocyanins, chlorophyll, and hydrogen peroxide were not significantly different; the contents of ETH were significantly decreased; and the contents of MeJA, SA, MDA, ABA, ETH, and IAA were significantly increased.

**Figure 9:**
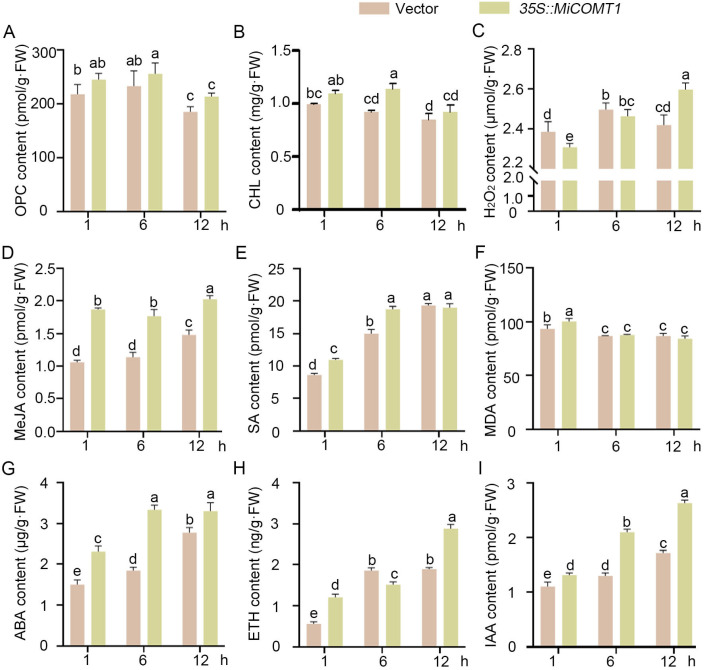
Overexpression of *MiCOMT1* confers salt tolerance in *N. benthamiana*: (**A**–**I**) *MiCOMT1* was overexpressed in *N. benthamiana* seedlings for three days, and then treated with 300 mmol·L^−1^ NaCl solution. Each experiment had three replicates and six *N. benthamiana* seedlings per replicate. Oligomeric Proanthocyanidin (OPC), chlorophyll (CHL), hydrogen peroxide (H_2_O_2_), methyl jasmonic acid (MeJA), salicylic acid (SA), malondialdehyde (MDA), abscisic acid (ABA), ethylene (ETH), and auxin (IAA) contents of *N. benthamiana* seedlings were treated as described. All data represent mean ± SE for three biological replications. Statistical significance was determined via Duncan’s test, and “a, b, c, d, and e” represent significant differences.

In order to evaluate salt tolerance, *N. benthamiana* seedlings were exposed to 300 mmol·L^−1^ NaCl when transient overexpression occurred for 3 days (Figure S3). The results showed that there was no significant difference between OPC and CHL contents after salt stress (Figure 9A,B), and the content of hydrogen peroxide also changed significantly (Figure 9C). In contrast to the control, the content of MeJA increased by more than 30% (Figure 9D), while the differences in the SA and MDA contents were fewer (Figure 9E,F). At 6 h, the ABA content increased by more than 145% compared to the control group (Figure 9G). Moreover, the contents of ETH and IAA were significantly higher in the *35S::MiCOMT1* group than in the control group at 1 h and 12 h (Figure 9H,I). Furthermore, using the control group as the x-axis and the *35S::MiCOMT1* group as the y-axis, simple linear regression analysis was performed using GraphPad Prism 9 software, and the result showed a significant positive correlation (y = 1.026x + 0.8835, R^2^ = 0.9944, *p* < 0.0001; Figure S4). It was confirmed that *MiCOMT1* activated phytohormone signals, affected the accumulation of anthocyanins and chlorophyll, and improved the salt tolerance of plants.

## 3. Discussion

In this study, for the first time, 18 *COMT* gene family members were systematically identified from the chromosome-level reference genome of mango. The number of COMT members in mango is smaller than that in *B. rapa* [4] and *G. max* [7]. The results of bioinformatic analysis showed that the 18 *MiCOMT* genes were divided into four groups, being unevenly distributed on 8 of the 20 chromosomes. As a comparison, 16 *COMT* genes were found in the *Citrullus lanatus* genome, and they were unevenly distributed on chromosomes 2, 7, 9, and 10. Some of the *ClCOMTs* were mapped to neighboring regions on the same chromosomes [9]. The rice *COMT* duplication gene pairs were concentrated on four chromosomes [8]. In addition, most *MiCOMTs* were found to be in the same group as *A. thaliana* and *M. esculenta*. Collinearity analysis showed that the *MiCOMT1* gene shares high homology with *A. thaliana*. 

The expression pattern of the *COMT* genes varies among different plant organs. In general, the expression of the *COMT* gene was higher in roots [7]. The *COMT* gene in *Larix gmelinii* was expressed in rhizomes and leaves, but the highest expression level was in stems [39]. The expression patterns of *OsCOMTs* in rice were tissue-specific, with high expression in the stem and low or no expression in the roots [8]. However, *MiCOMTs* are highly expressed in mango flowers. These results suggest that *COMT* exhibits different functions in different plants. It is possible that the different structures and growth patterns of woody and herbaceous plants lead to different expression patterns of *COMT* in different plant organs.

Plants are often subjected to various biotic and abiotic stresses during their growth and development, such as high-temperature stress [40], drought stress [41], pathogen stress [42], and salt stress [43]. The plant response to stresses involves complex regulatory mechanisms. For example, *OsASR6* in rice enhances tolerance to salt stress [44]; in Arabidopsis, *MBF1c* is a highly conserved transcriptional co-activator that plays an important role in the heat stress response (HSR) [45]. It is known that *COMT* gene expression is responsive to salt [46], drought [15], and pathogens [47]. However, *COMT* genes were down-regulated in salt-treated roots of rice [48]. The expression of *Ligusticum chuanxiong LcCOMT* genes remained unchanged after salt stress [49]. The expression of the *MiCOMT* genes was significantly increased after drought stress in mango. The discrepancy between these results might illustrate a response variation among different stress conditions and plant species. Meanwhile, ABA and SA play important roles in plant stress resistance, and SA regulates the heat and photo-oxidative stress of leaves [50]. ABA functions as a central integrator that links and reprograms the complex developmental process and salt stress, and especially osmotic stress, and adaptive signaling cascades in plants [51]. In mango, *X. campestris* pv. *mangiferaeindicae* and *C. gloeosporioides* are the most representative bacterium and pathogenic fungus of mango, respectively [52]. We analyzed the *MiCOMT* genes’ expression levels during infection of the leaves with *X. campestris* pv. *mangiferaeindicae* and *C. gloeosporioides* at 0–72 h. The expression of *MiCOMT* genes was significantly up-regulated upon *X. campestris* pv. *mangiferaeindicae* infection. This may be because *C. gloeosporioides* is a fungus and *X. campestris* pv. *mangiferaeindicae* is a bacterium, and the mechanism of infection is different, resulting in different resistance patterns in mango. Similarly, the expression of *HbCOMT* increased rapidly after *Hevea brasiliensis* suffered from tapping panel dryness [53]. These results suggest that *COMT* may participate in the plant stress response.

Plants absorb various nutrients from the soil during growth and development [54], but high concentrations of salt ions in the soil inhibit the growth of plants [55]. So, it is important to study the molecular mechanisms of salt tolerance in plants [56]. The plant COMT protein is widely recognized as a stress response protein under salt stress [57]. Impaired photosynthetic systems could impede photosynthetic electron transport, resulting in the accumulation of excessive ROS (H_2_O_2_) under salt stress conditions [58]. This excess reactive oxygen species activates anthocyanins, which protect plants from damage from reactive oxygen species [59]. At the same time, it integrates and coordinates various phytohormones’ levels and functions, such as ABA [60], SA, MeJA, and ETH [51]. In this study, low H_2_O_2_ concentrations were detected in the *MiCOMT1*-overexpressing lines after 1–6 h salt treatment. Compared with the control group, the H_2_O_2_ content at 12 h and MDA at 1 h were significantly increased. When tomato seeds were tested with salt treatment, H_2_O_2_ and MDA contents increased by approximately 66% and 33%, respectively [61]. This was consistent with the concentration of H_2_O_2_ and MDA detected in the *SlCOMT1*-overexpression line after salt treatment [62]. Studies have shown that application of MeJA has a positive effect on antioxidant enzymes to protect plant under abiotic stress conditions [63]. MeJA protects wheat seedlings from salt stress by inhibiting excess reactive oxygen species [64]. In this study, the content of MeJA increased by more than 30%. Many studies have shown that SA is involved in plant salt stress [65,66]. Moreover, the contents of SA were significantly higher in the *35S::MiCOMT1* group than in the control group at 1–6 h. ABA plays an important role in salt stress by regulating stomatal opening or closure [67]. In the salt treatment of *N. benthamiana* overexpressing *MiCOMT1*, after 6 h, the ABA content increased by more than 145% compared to the control group. In leaves and roots, the *CrCOMT* gene positively responded to ABA treatment [14]. The content of the *MiCOMT1* gene in mango increased significantly at 24–72 h, suggesting that *COMT* may be involved in the ABA-dependent stress tolerance pathway. In rice, transcriptome analysis has shown that the expression of IAA-responsive genes is up-regulated under salt stress condition [68]. Exogenously applied ethylene can significantly increase salt stress tolerance in Arabidopsis seedlings [69]. Moreover, the contents of ETH and IAA were significantly higher in the *35S::MiCOMT1* group than in the control group at 1 h and 12 h. In addition, other phytohormones were also increased after salt stress compared to the control group. Thus, it is likely that *MiCOMT1* might be a positive regulator of the response to salt by influencing the expression of stress-responsive genes.

## 4. Materials and Methods

### 4.1. Plant Materials and Growth Conditions

The Danzhou Mango Germplasm Resource Bank of the Ministry of Agriculture and Rural Affairs provided various tissues and annual seedlings such as roots, young leaves, young stems, flowers, fruits, and seeds, and the variety was “Guifei”. Seedlings were potted in soil (professional growing mix, soil/vermiculite = 2:1) in a greenhouse at 28 °C under a 16/8 h light/dark photoperiod [70,71,72].

Mango leaves were sprayed with a 2 × 10^7^ conidia·mL^−1^ suspension of *C. gloeosporioides*, 2 × 10^6^ cfu·mL^−1^ *X. campestris* pv. *Mangiferaeindicae* suspension, 5 mmol·L^−1^ salicylic acid solution, and 5 mmol·L^−1^ abscisic acid solution, until the leaves were all wet, and then protected from light. And the mango seedlings were irrigated with 300 mmol·L^−1^ NaCl and 30% PEG6000 [73,74] until the soil in the pot was completely soaked. We observed the mango leaves at 0, 3, 6, 12, 24, 48, and 72 h (Figure S5). Then, we quickly froze the samples in liquid nitrogen and stored them in a refrigerator at −80 °C. Each treatment was repeated in three replicates [75].

### 4.2. Identification Analysis of MiCOMT Genes

The chromosome mapping information of the *COMT* gene family in mango (Taxonomy ID 29780) was gained from the NCBI (https://www.ncbi.nlm.nih.gov/, accessed on 12 November 2022) database, and the map was drawn using Tbtools v1.098768 software [76]. A total of 18 *COMT* genes were retrieved after performing BLAST, as well as identifying and removing identical genes in the mango genome (genome assembly: CATAS_Mindica_2.1, NCBI RefSeq assembly: GCF_011075055.1), based on the fact that they have the same domain, named *MiCOMT1* to *MiCOMT18*. Syntenic analysis of *COMT* genes in mango, *Arabidopsis thaliana*, was conducted by the Tbtools v1.098768 software, which embeds Mcscan (https://github.com/wyp1125/MCScanX, accessed on 15 November 2022) software with the default parameters. The genome of *Arabidopsis thaliana* was downloaded from the Arabidopsis (https://www.ncbi.nlm.nih.gov/datasets/taxonomy/3702/, accessed on 20 November 2022) databases. The Ka value, Ks value, and Ka/Ks ratios for the paralog *COMT* gene pairs were calculated using Tbtools, and the rate of divergence was calculated by using the following formula: T = Ks/2r, where Ks represents the synonymous substitutions per site and r is the rate of divergence. We evaluated the values of Ks (synonymous) and Ka (nonsynonymous) as well as the ratio of Ka/Ks. Ka/Ks < 1, Ka/Ks = 1, and Ka/Ks > 1 generally indicate negative, neutral, and positive selection, respectively [76,77,78].

### 4.3. Phylogenetic and Motif Analysis of MiCOMT Genes

The protein sequences of *COMT* from *M. indica*, *A. thaliana*, *M. esculenta*, *N. tabacum*, *S. lycopersicum*, *O. sativa*, and *P. bretschneideri* (Table S1) were analyzed with MEGA 11 software, based on multiple sequence alignment; the phylogenetic tree was constructed according to the method of Salih and colleagues [79] and Li and colleagues [80], and using the neighbor-joining (NJ) method in MEGA 11 software. The robustness of each node in the tree was determined using 1000 bootstrap replicates, and the default parameter for the remaining parameters was selected.

The gene structures of the *COMT* gene family members in mango were visualized using the mango genome’s annotation information using Tbtools v1.098768 software. The classic mode motifs of COMT proteins were analyzed by the MEME website (http://meme-suite.org; accessed on 30 November 2022) and Tbtools v1.098768 software [81,82].

### 4.4. RNA Isolation and Quantitative Real-Time PCR (qRT-PCR) 

We ground the plant material into a powder in liquid nitrogen and extracted it using an RNA extraction kit (centrifugal column type) (TIANGEN, Beijing, China) [83]. We used QuantStudio 6 Flex (Applied Biosystems, Waltham, MA, USA) to determine the expression of the gene and 2^−ΔΔCT^ to calculate it [52,73]. The qRT–PCR primers of the *MiCOMT* genes were designed via Primer3 Plus (https://www.bioinformatics.nl/cgi–bin/primer3 plus/primer3 plus.cgi, accessed on 14 March 2023) to determine the specific primers (Table S10) [84].

### 4.5. Construction of the Expression Vectors and Transient Expression

The full-length coding regions of *MiCOMT1* were PCR-amplified using *Pfu* polymerase with primers (Table S11) and cloned into an overexpression pEGAD vector via appropriate restriction enzyme digestion with *Bam* HⅠ, *Hind* III, and T4 DNA ligase [85]. The recombinant plasmid was named *35S::MiCOMT1*. The recombinant plasmid and control vector (vector) were transformed into *Agrobacterium* GV3101 by the heat shock method [85]. 

The *A. tumefaciens* suspension was used to infect the leaves of *N. benthamiana*. *N. benthamiana* leaves were injected with *Agrobacterium* GV3101 for 3 days. Next, for the 300 mmol·L^−1^ NaCl treatment in *N. benthamiana*, the plants were photographed, and the leaves were harvested for physiological index and gene expression analyses.

### 4.6. Determination of Related Physiological and Biochemical Parameters

According to the experimental method of Chiriboga and colleagues [86], anthocyanins were extracted by a mixed solution of HCl with a solvent of 1.5 mol/L and a volume fraction of 95% ethyl alcohol (volume ratio of 15:85), and calculated with the following formula: OPC contents (nmol/g·FW) =$\;(\frac{\left({OD}_{530}-{OD}_{620}\right)-0.1\times \left({OD}_{650}-OD_{620}\right)}{4.62\;\times \;10^{6}}\times \frac{V}{m}\times 1,000,000)$. This study referred to Zhang’s method to extract chlorophyll with 95% alcohol in the dark for 24 h, and this was calculated with the following formula: CHL content (mg/g·FW) =$\;\frac{\left(17.32\times {OD}_{649}+7.18\times {OD}_{665}\right)}{1000}\times \frac{V}{m}$ [14].

Following the method of Wei and colleagues [87], the leaves were flash-frozen in liquid nitrogen and ground to a very fine powder. The catalase activity was extracted from 0.1 g powder using phosphate-buffered solution (PBS, pH 7.4, 0.15 mol·L^−1^) on ice. Catalase activity in the supernatant was determined by quantifying the decomposition of H_2_O_2_. The endogenous H_2_O_2_ level in plant leaves was quantified using 0.1% titanium sulphate at a wavelength of 410 nm. 

The leaves were flash-frozen in liquid nitrogen and ground to a very fine powder. The endogenous phytohormones were extracted from 0.1 g powder using phosphate-buffered solution (PBS, pH 7.4, 0.15 mol·L^−1^) on ice. Extraction and content determination of MeJA, SA, MDA, ABA, ETH, and IAA were performed using the ELISA Kit (Jiangsu Meimian Industrial, Yancheng, China), according to Zhang and colleagues’ method [88], and the article numbers sequentially are MM-6291801, MM-3372201, MM-200901, MM-118501, MM-088801, and MM-095301. 

### 4.7. Statistical Analysis

All the data were processed using GraphPad Prism 9 and IBM SPSS Statistics 26, and we used the one-way ANOVA test for analysis. Equal variance was assumed and Duncan’s multiple range test was used for mean comparisons. In this study, lowercase letters indicate a significant difference (*p* < 0.05). “#” represents significantly up-regulated gene expression. “*” represents significantly down-regulated gene expression.

## 5. Conclusions

In the current study, a total of 18 *COMT* genes were identified from the mango genome, which had high sequence similarity and a close evolutionary relationship with Arabidopsis gene homologs. Through tissue-specific expression analysis, it was found that the expression level was the highest in mango flowers. In addition, we reported a mango *COMT* gene (*MiCOMT1*) that plays a role in regulating salt stress tolerance. We also confirmed that *MiCOMT1* is a positive regulator of plant salinity tolerance to abiotic stresses. To our knowledge, *MiCOMT1* is the first *COMT* family gene cloned in the mango. The findings presented here could be exploited to benefit mango production in favorable and unfavorable environmental conditions. This paper can provide a reference for researchers engaged in studies on gene function and mechanism identification in mango and other fruits’ disease resistance. It also provides ideas for researchers engaged in *COMT* gene research.

## Data Availability

Data contained within the article.

## Supplementary Materials

The following supporting information can be downloaded at https://www.mdpi.com/article/10.3390/ijms25052639/s1.
