# Supplementary material for: Caffeic Acid O-Methyltransferase Gene Family in Mango (Mangifera indica L.) with Transcriptional Analysis under Biotic and Abiotic Stresses and the Role of MiCOMT1 in Salt Tolerance
Source: Int J Mol Sci. 2024 Feb 24;25(5):2639. doi: 10.3390/ijms25052639 (PMC10931984; doi:10.3390/ijms25052639)
Supplement: Supplementary file 1 [file ijms-25-02639-s001.zip › Figure S1 S2 S3 S4 S5.pdf]

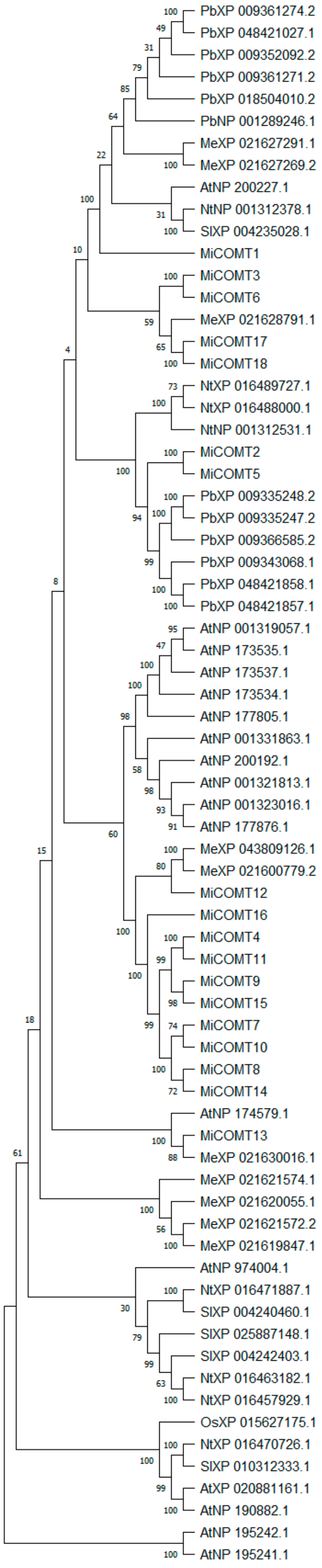
PbXP 009361274.2
PbXP 048421027.1
PbXP 009352092.2
PbXP 009361271.2
PbXP 018504010.2
PbNP 001289246.1
MeXP 021627291.1
MeXP 021627269.2
AtNP 200227.1
NtNP 001312378.1
SlXP 004235028.1
MiCOMT1
MiCOMT3
MiCOMT6
MeXP 021628791.1
MiCOMT17
MiCOMT18
NtXP 016489727.1
NtXP 016488000.1
NtNP 001312531.1
MiCOMT2
MiCOMT5
PbXP 009335248.2
PbXP 009335247.2
PbXP 009366585.2
PbXP 009343068.1
PbXP 048421858.1
PbXP 048421857.1
AtNP 001319057.1
AtNP 173535.1
AtNP 173537.1
AtNP 173534.1
AtNP 177805.1
AtNP 001331863.1
AtNP 200192.1
AtNP 001321813.1
AtNP 001323016.1
AtNP 177876.1
MeXP 043809126.1
MeXP 021600779.2
MiCOMT12
MiCOMT16
MiCOMT4
MiCOMT11
MiCOMT9
MiCOMT15
MiCOMT7
MiCOMT10
MiCOMT8
MiCOMT14
AtNP 174579.1
MiCOMT13
MeXP 021630016.1
MeXP 021621574.1
MeXP 021620055.1
MeXP 021621572.2
MeXP 021619847.1
AtNP 974004.1
NtXP 016471887.1
SlXP 004240460.1
SlXP 025887148.1
SlXP 004242403.1
NtXP 016463182.1
NtXP 016457929.1
OsXP 015627175.1
NtXP 016470726.1
SlXP 010312333.1
AtXP 020881161.1
AtNP 190882.1
AtNP 195242.1
AtNP 195241.1

Figure S1. Phylogenetic relationship of COMTs in different species using complete protein sequences. The neighbor–joining (NJ) phylogenetic tree was constructed with the Poisson model using MEGA 11 software. The tree was generated from an amino acid sequence alignment of *Mangifera indica (Mi)* (18 members), *Arabidopsis thaliana (At)* (17 members), *Manihot esculent (Me)* (10 members), *Nicotiana tabacum Nt*) (8 members), *Solanum lycopersicum (Sl)* (5 members), *Oryza sativa (Os)* (1 member) and *Pyrus bretschneideri (Pb)* (12 members). The number on the branch indicates Bootstrap.

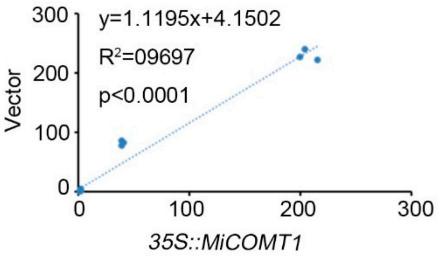

Figure S2. Linear regression of MiCOM gene 35S::MiCOMT1 with vector after 3 days of *N. benthamiana* overexpression. using the control group as x axis and *35S::MiCOMT1* group as y axis, simple linear regression analysis was performed by GraphPad Prism software, and the result showed a significant positive correlation (y=1.1195x+4.1502, R^2=0.9697, p<0.0001).

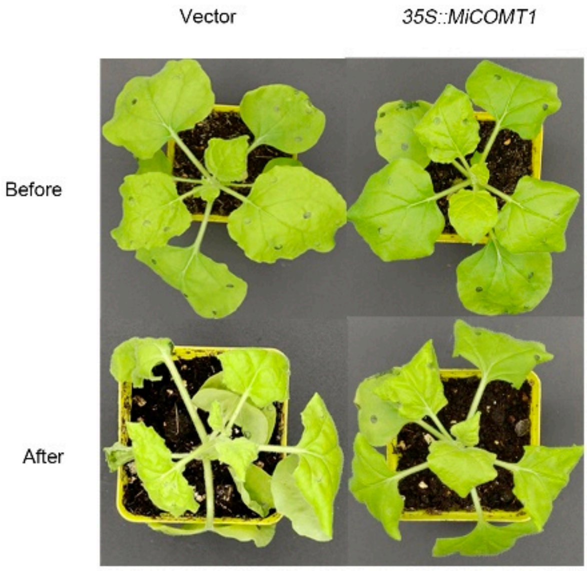

Figure S3. Morphology of *N. benthamiana* seedlings after 3 days of overexpression of *MiCOMT* before and after irrigation with 300 mM NaCl solution.

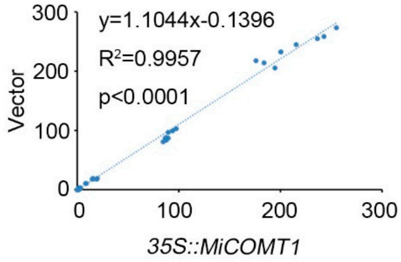

Figure S4. MiCOMT1 was overexpressed in *N. benthamiana* seedlings for three days, and then treated with 300 mM NaCl solution, Linear regression of 35S::MiCOMT1 with vector. using the control group as x axis and *35S::MiCOMT1* group as y axis, simple linear regression analysis was performed by GraphPad Prism software, and the result showed a significant positive correlation (y=1.1044x-0.1396, R^2=0.9957, p<0.0001).

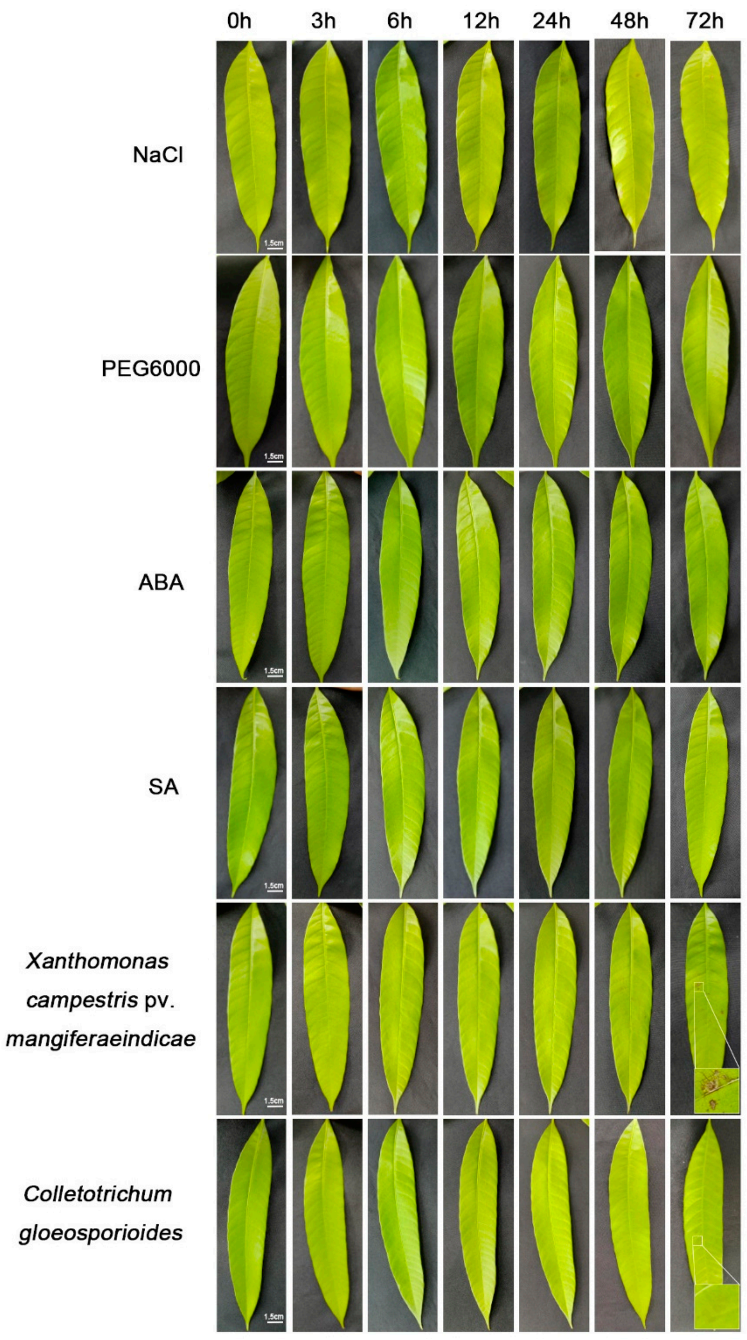

Figure S5. Phenotypes of mango leaves at different time periods under different treatments. Mango seedlings were treated with 300 mmol·L-1 NaCl (A) and 30% PEG6000 (B) for 0, 3, 6, 12, 24, 48, and 72 h. Mango seedlings leaves were treated with 5 mmol·L^{-1} ABA (C) and SA (D) and 2 × 10^6 mL^{-1} suspension of *Xanthomonas campestris* pv. *mangiferaeindicae* bacteric (E) and 2 × 10^7 mL^{-1} suspension of *Colletotrichum gloeosporioides* conidia (F) for 0, 3, 6, 12, 24, 48, and 72 h.
