# Supplementary material for: Caffeic Acid O-Methyltransferase Gene Family in Mango (Mangifera indica L.) with Transcriptional Analysis under Biotic and Abiotic Stresses and the Role of MiCOMT1 in Salt Tolerance
Source: Int J Mol Sci. 2024 Feb 24;25(5):2639. doi: 10.3390/ijms25052639 (PMC10931984; doi:10.3390/ijms25052639)
Supplement: Supplementary file 1 [file ijms-25-02639-s001.zip › Table S1.pdf]

Table S1. COMT protein ID of each species.

Species name	Sequence protein ID
Mangifera indica L.	>MiCOMT1
	>MiCOMT2
	>MiCOMT3
	>MiCOMT4
	>MiCOMT5
	>MiCOMT6
	>MiCOMT7
	>MiCOMT8
	>MiCOMT9
	>MiCOMT10
	>MiCOMT11
	>MiCOMT12
	>MiCOMT13
	>MiCOMT14
	>MiCOMT15
	>MiCOMT16
	>MiCOMT17
	>MiCOMT18
Nicotiana tabacum	>NP_001312531.1
	>NP_001312378.1
	>XP_016489727.1
	>XP_016488000.1
	>XP_016471887.1
	>XP_016470726.1
	>XP_016463182.1
	>XP_016457929.1
Arabidopsis thaliana	>XP_020881161.1
	>NP_177805.1
	>NP_195242.1
	>NP_200227.1
	>NP_001321813.1
	>NP_974004.1
	>NP_001323016.1
	>NP_173537.1
	>NP_001331863.1
	>NP_174579.1
	>NP_001319057.1
	>NP_173534.1
	>NP_190882.1
	>NP_173535.1
	>NP_177876.1
	>NP_200192.1
	>NP_195241.1
Pyrus bretschneideri	>NP_001289246.1
	>XP_009366585.2
	>XP_048421716.1

	>XP_009361274.2
	>XP_018504010.2
	>XP_009361271.2
	>XP_048421027.1
	>XP_009352092.2
	>XP_009343068.1
	>XP_009335248.2
	>XP_009335247.2
	>XP_048421858.1
	>XP_048421857.1
Manihot esculenta	>XP_021628791.1
	>XP_021620055.1
	>XP_021630016.1
	>XP_021627291.1
	>XP_021627269.2
	>XP_021621574.1
	>XP_021621572.2
	>XP_021619847.1
	>XP_043810147.1
	>XP_043809126.1
	>XP_021600779.2
Solanum lycopersicum	>XP_010312333.1
	>XP_004240460.1
	>XP_004235028.1
	>XP_025887148.1
	>XP_004242403.1
Oryza sativa	>XP_015627175.1
