# Supplementary material for: Caffeic Acid O-Methyltransferase Gene Family in Mango (Mangifera indica L.) with Transcriptional Analysis under Biotic and Abiotic Stresses and the Role of MiCOMT1 in Salt Tolerance
Source: Int J Mol Sci. 2024 Feb 24;25(5):2639. doi: 10.3390/ijms25052639 (PMC10931984; doi:10.3390/ijms25052639)
Supplement: Supplementary file 1 [file ijms-25-02639-s001.zip › Table S10.pdf]

Table S10. Primer sequences for qRT-PCR

Gene name	Forward primer（5′—3′）	Reverse primer (5′—3′)
MiCOMT1	GGCAAAGATCCCAGATTCAA	CAAAGATGGAGCATCGTCAA
MiCOMT2	TTCCAAAAGGTGATGCCATT	CGCTTGAATCATCAAAAGCA
MiCOMT3	ATCCGACGTAGTGGCTTTTG	AAGCCCTTTGAATCCTTGGT
MiCOMT4	ATTGGGGTGATGAAGACTGC	TCCGGATTCCAAAGCTAATG
MiCOMT5	CATGACCTTGAAAGCAGCAA	GCTAGCGAGAAGCCTAAGCA
MiCOMT6	ATCCGACTTTGTGGCACTTC	GCCATTGCCTTGTTGAAAAT
MiCOMT7	GATCCAAAGCACCACCAGTT	CAACATGCTCAACACCTGCT
MiCOMT8	ATTGGGGTGATGAAGACTGC	CCGAAGCTAGTGCCTCAAAC
MiCOMT9	ATTGGGGTGATGAAGACTGC	TCCGGATTCCAAAGCTAATG
MiCOMT10	GCACCCATTTGCAAGTTTTT	TGGTGCTTTGGATCCTTTTC
MiCOMT11	ATTGGGGTGATGAAGACTGC	TCCGGATTCCAAAGCTAATG
MiCOMT12	GTTCTTCCCATGGTCCTCAA	CGGCACCATACACTCTCTCA
MiCOMT13	CGATCATGATGGTGGTCAAG	CTCATCCCATACGCCAAGTT
MiCOMT14	ATTGGGGTGATGAAGACTGC	ATCCGGATTCCGAAGCTAAT
MiCOMT15	ATTGGGGTGATGAAGACTGC	TCCGGATTCCAAAGCTAATG
MiCOMT16	GCACCTCCAAAATCAGGTGT	CTTTCCCATGGTTTGGAAGA
MiCOMT17	CTTTGCCAATGTGTTGAACG	AGAGCCTCTCAATGCTTCCA
MiCOMT18	ATTCCAATAACAGCGGATGC	TTTGCCTCCATGCATAATCA
MiActin1	GGCAAGTCTGGTGCCAG	ACGGTATCTATCTCTTCG
