# Supplementary material for: Caffeic Acid O-Methyltransferase Gene Family in Mango (Mangifera indica L.) with Transcriptional Analysis under Biotic and Abiotic Stresses and the Role of MiCOMT1 in Salt Tolerance
Source: Int J Mol Sci. 2024 Feb 24;25(5):2639. doi: 10.3390/ijms25052639 (PMC10931984; doi:10.3390/ijms25052639)
Supplement: Supplementary file 1 [file ijms-25-02639-s001.zip › Table S11.pdf]

Table S11. The primers used in this study.

Primer name	Sequence (5′-3′)	Expected length /bp
MiCOMT1-F	ATGGGTTCCACAGGAGAAAC	1101
MiCOMT1-R	TCAAACACTCTTAATAAATT	
EG-MiCOMT1-F	CCCAAGCTTGGGATGGGTTCCACAGGAGAAAC	1113
EG-MiCOMT1-R	CGCGGATCCGCGTCAAACACTCTTAATAAATT	
