# Supplementary material for: Caffeic Acid O-Methyltransferase Gene Family in Mango (Mangifera indica L.) with Transcriptional Analysis under Biotic and Abiotic Stresses and the Role of MiCOMT1 in Salt Tolerance
Source: Int J Mol Sci. 2024 Feb 24;25(5):2639. doi: 10.3390/ijms25052639 (PMC10931984; doi:10.3390/ijms25052639)
Supplement: Supplementary file 1 [file ijms-25-02639-s001.zip › Table S2.pdf]

Table S2. The Ka, Ks and Ka/Ks values of MiCOMT genes.

Species 1	Species 2	Ka	Ks	Ka/Ks
MiCOMT1	MiCOMT3	2.850	1.828	1.559070343
MiCOMT1	MiCOMT4	2.734	1.859	1.47085514
MiCOMT2	MiCOMT4	2.162	2.143	1.008566625
MiCOMT3	MiCOMT4	2.546	1.942	1.310986431
MiCOMT1	MiCOMT5	2.763	2.596	1.064230843
MiCOMT2	MiCOMT5	2.797	2.112	1.32456417
MiCOMT3	MiCOMT5	2.064	2.569	0.803166722
MiCOMT4	MiCOMT5	0.768	1.747	0.439549487
MiCOMT3	MiCOMT6	0.049	0.196	0.251163163
MiCOMT4	MiCOMT6	2.706	1.949	1.387915856
MiCOMT5	MiCOMT6	2.171	2.537	0.855665051
MiCOMT2	MiCOMT7	1.869	2.304	0.811225355
MiCOMT5	MiCOMT7	3.184	2.019	1.5764554
MiCOMT2	MiCOMT8	1.912	2.514	0.760450363
MiCOMT4	MiCOMT8	3.001	3.180	0.943780048
MiCOMT7	MiCOMT8	0.020	0.043	0.460714296
MiCOMT1	MiCOMT9	2.640	1.729	1.527258946
MiCOMT2	MiCOMT9	2.068	2.219	0.931793195
MiCOMT3	MiCOMT9	2.384	1.999	1.192293689
MiCOMT4	MiCOMT9	0.029	0.056	0.510050419
MiCOMT5	MiCOMT9	0.759	1.776	0.427068521
MiCOMT6	MiCOMT9	2.429	2.032	1.195447505
MiCOMT2	MiCOMT10	1.852	2.968	0.62392517
MiCOMT7	MiCOMT10	0.010	0.030	0.327800658
MiCOMT8	MiCOMT10	0.015	0.048	0.311867084
MiCOMT1	MiCOMT11	2.734	1.765	1.549198233
MiCOMT2	MiCOMT11	2.162	2.060	1.049607358
MiCOMT3	MiCOMT11	2.528	1.915	1.320296826
MiCOMT4	MiCOMT11	0.001	0.013	0.09685975
MiCOMT5	MiCOMT11	0.770	1.769	0.435036835
MiCOMT6	MiCOMT11	2.683	1.922	1.396140924
MiCOMT7	MiCOMT11	3.017	2.885	1.045897816
MiCOMT8	MiCOMT11	3.035	2.836	1.070147044
MiCOMT9	MiCOMT11	0.030	0.061	0.493161421
MiCOMT10	MiCOMT11	3.099	3.080	1.006153799
MiCOMT1	MiCOMT12	2.326	1.707	1.362650252
MiCOMT4	MiCOMT12	0.558	1.201	0.464906521
MiCOMT5	MiCOMT12	0.488	2.169	0.22509402
MiCOMT6	MiCOMT12	2.789	2.533	1.101367252
MiCOMT7	MiCOMT12	3.129	2.507	1.247937046
MiCOMT9	MiCOMT12	0.570	1.186	0.480755628
MiCOMT11	MiCOMT12	0.561	1.141	0.491422216
MiCOMT1	MiCOMT13	1.746	2.200	0.793758285
MiCOMT2	MiCOMT13	3.180	2.442	1.301998063
MiCOMT3	MiCOMT13	2.842	2.374	1.197230649
MiCOMT12	MiCOMT13	3.072	2.712	1.133028744
MiCOMT4	MiCOMT14	2.890	3.120	0.926227981
MiCOMT5	MiCOMT14	3.218	2.060	1.562269705
MiCOMT7	MiCOMT14	0.015	0.052	0.284502489
MiCOMT9	MiCOMT14	2.671	3.230	0.827060476
MiCOMT10	MiCOMT14	0.012	0.057	0.217631612
MiCOMT11	MiCOMT14	2.919	2.798	1.043563004
MiCOMT12	MiCOMT14	3.168	2.714	1.166939086
MiCOMT1	MiCOMT15	2.913	1.708	1.705673035
MiCOMT2	MiCOMT15	2.092	2.321	0.90116819
MiCOMT3	MiCOMT15	2.468	2.083	1.184782404
MiCOMT4	MiCOMT15	0.022	0.056	0.398605591

MiCOMT5	MiCOMT15	0.766	1.725	0.443739626
MiCOMT6	MiCOMT15	2.552	2.125	1.201102009
MiCOMT7	MiCOMT15	2.750	2.825	0.973350773
MiCOMT8	MiCOMT15	2.867	2.781	1.031074325
MiCOMT9	MiCOMT15	0.011	0.008	1.330735489
MiCOMT10	MiCOMT15	2.918	2.802	1.041132399
MiCOMT11	MiCOMT15	0.024	0.061	0.389867997
MiCOMT12	MiCOMT15	0.563	1.221	0.460933139
MiCOMT14	MiCOMT15	2.773	2.745	1.010362335
MiCOMT1	MiCOMT16	2.762	1.826	1.512630406
MiCOMT3	MiCOMT16	3.065	2.266	1.352664292
MiCOMT4	MiCOMT16	1.602	1.934	0.828506353
MiCOMT5	MiCOMT16	1.333	2.086	0.639071404
MiCOMT6	MiCOMT16	3.229	1.927	1.675399698
MiCOMT7	MiCOMT16	2.209	2.275	0.97092603
MiCOMT8	MiCOMT16	2.265	2.247	1.00779079
MiCOMT9	MiCOMT16	1.563	1.929	0.810302225
MiCOMT10	MiCOMT16	2.252	2.132	1.056425573
MiCOMT11	MiCOMT16	1.605	1.868	0.859312974
MiCOMT12	MiCOMT16	0.639	1.280	0.499185701
MiCOMT13	MiCOMT16	2.531	3.076	0.822720818
MiCOMT14	MiCOMT16	2.196	2.675	0.821148148
MiCOMT15	MiCOMT16	1.567	2.156	0.726875653
MiCOMT1	MiCOMT17	3.197	2.002	1.597176017
MiCOMT3	MiCOMT17	1.287	1.977	0.651290027
MiCOMT4	MiCOMT17	2.567	2.851	0.900434181
MiCOMT5	MiCOMT17	1.993	1.909	1.044397272
MiCOMT6	MiCOMT17	1.298	2.061	0.629679327
MiCOMT9	MiCOMT17	2.620	2.214	1.183577403
MiCOMT11	MiCOMT17	2.561	2.709	0.945314557
MiCOMT12	MiCOMT17	2.449	2.748	0.890953977
MiCOMT15	MiCOMT17	2.631	2.471	1.064949531
MiCOMT16	MiCOMT17	2.003	1.659	1.20752704
MiCOMT1	MiCOMT18	2.717	2.472	1.098993997
MiCOMT3	MiCOMT18	1.306	1.996	0.654443269
MiCOMT5	MiCOMT18	2.078	2.120	0.980139669
MiCOMT6	MiCOMT18	1.317	2.414	0.545399763
MiCOMT8	MiCOMT18	3.231	3.195	1.011333015
MiCOMT14	MiCOMT18	3.228	2.762	1.168569004
MiCOMT16	MiCOMT18	2.198	1.842	1.193132604
MiCOMT17	MiCOMT18	0.048	0.118	0.405187566
MiCOMT9	MiCOMT14	2.671	3.230	0.827060476
