# Supplementary material for: Caffeic Acid O-Methyltransferase Gene Family in Mango (Mangifera indica L.) with Transcriptional Analysis under Biotic and Abiotic Stresses and the Role of MiCOMT1 in Salt Tolerance
Source: Int J Mol Sci. 2024 Feb 24;25(5):2639. doi: 10.3390/ijms25052639 (PMC10931984; doi:10.3390/ijms25052639)
Supplement: Supplementary file 1 [file ijms-25-02639-s001.zip › Table S3 S4 S5 S6 S7 S8 S9 .pdf]

Table S3. The expression of *MiCOMT* genes in different mango tissues.

Gene name	Root	Stem	Leaf	Flower	Fruit	Seed
MiCOMT1	1	123.2471988	2.850575784	89.82169583	108.5836837	8.935744034
MiCOMT2	1	317.0713439	458.1979582	210.3618796	58.82088069	402.9105993
MiCOMT3	1	65.04110333	9.337344347	131.9976783	226.7452131	0.654665648
MiCOMT4	1	0.009328593	0.007808855	59.77106951	3.554934987	7.140132523
MiCOMT5	1	2.509607731	2.238751339	97.12219182	24.72314633	3.323775762
MiCOMT6	1	4.525429774	2.928986792	29.48095438	192.8858627	64.53375034
MiCOMT7	1	2.3590982	2.445036423	188.5091515	391.8775415	361.3978599
MiCOMT8	1	0.025782479	0.006180011	34.35787594	0.594736425	1.073260699
MiCOMT9	1	0.045458982	0.002973301	132.881554	5.587210376	5.034077216
MiCOMT10	1	0.006710298	0.10232162	186.7255639	1	1
MiCOMT11	1	0.003176079	0.002300162	36.02301274	1.473591843	3.619616543
MiCOMT12	1	0.637965266	0.640078193	60.9533168	21.92565265	22.50200275
MiCOMT13	1	18.52169375	4.462155873	83.57537111	520.6577529	134.6019539
MiCOMT14	1	0.013217268	0.002199057	53.66582032	1.378190113	5.002704126
MiCOMT15	1	0.054600531	0.014616869	84.95160714	5.697366568	1.533677869
MiCOMT16	1	162981.5909	1.871359104	524.3980679	102.2238915	40.46738996
MiCOMT17	1	124.2570448	48.09190409	28.3929578	24.69687002	0.000299529
MiCOMT18	1	35794.6893	37290.49322	3429.048466	67129.43113	8.106825361

Table S4. Expression patterns of *MiCOMTs* genes under salt stresses.

Gene name	0h	3h	6h	12h	24h	48h	72h
MiCOMT1	1	0.619303439	1.750754797	33.85274503	24.44306941	39.62820692	39.57837912
MiCOMT2	1	0.120469128	0.781973956	0.107841814	7.516283881	0.000299377	0.00153115
MiCOMT3	1	0.002244059	19.45146546	84.21928635	36.95235878	7.666040653	17.49694934
MiCOMT4	1	0.000416031	0.607205025	0.634226904	0.098776307	0.002013622	0.190771552
MiCOMT5	1	0.902669327	14.97429734	1.296686972	2.343784415	0.088669325	2.948528247
MiCOMT6	1	0.100221433	40.10019546	167.4918495	6.002997998	2.25089365	2.325537598
MiCOMT7	1	0.006855446	0.107355025	0.238824631	0.070110856	0.093604831	0.00954372
MiCOMT8	1	0.831923342	2.264562378	0.017954972	0.002863114	0.004418208	0.000502051
MiCOMT9	1	3.702530597	115.8629042	12.90316925	0.004443894	0.000431387	0.000110859
MiCOMT10	1	0.098949795	0.004894588	0.00555774	0.041165744	0.008172718	0.0000360787
MiCOMT11	1	23.60503048	1.566656124	116.0286094	0.136954135	0.025040037	0.024750381
MiCOMT12	1	0.007032143	1.518964738	15.83397241	57.92022004	6.459820101	0.089746373
MiCOMT13	1	0.229563605	38.45796367	31.62841184	1.2588266	128.073352	2.288183985
MiCOMT14	1	0.000645075	0.629586962	11.5085287	13.34919205	0.5891942	0.060102198
MiCOMT15	1	0.002476	1.278253	19.34339	10.24685	2.459487	0.091853454
MiCOMT16	1	18.27062	37.32463	163.5574	35.77125	0.052334	0.000150434
MiCOMT17	1	0.243093	0.294979	13.37145	4.161918	0.566167	0.00000361862
MiCOMT18	1	8.686435	2.909295	148.1744	319.7003	0.002278	0.003627352

Table S5. Expression patterns of MiCOMTs genes under drought stresses.

Gene name	0h	3h	6h	12h	24h	48h	72h
MiCOMT1	1	3.423901322	7.483985101	6.090576647	2.275264	5.757653	12.50520552
MiCOMT2	1	3.237491383	3.042562536	2.860184464	25.46391	32.35293	4.562302528
MiCOMT3	1	1.997957119	1.532812989	3.447174611	1.05128	8.347136	6.187902565
MiCOMT4	1	3.923292698	75.470918	439.0941288	539.1548	21.0657	3.069129755
MiCOMT5	1	7.711458145	18423.41279	589393.2146	1801245	3094.811	1156.370774
MiCOMT6	1	2.488530839	3.868582699	1163.513929	3098.862	18.88286	25.70858024
MiCOMT7	1	1.760664649	90.96864859	1251.987749	947.8888	30.91543	13.86597708
MiCOMT8	1	0.913232532	19.57172491	227.1226423	276.561	65.8234	43.79313737
MiCOMT9	1	1.063202043	43.29105365	297.6057217	389.1955	65.64692	4.710355768
MiCOMT10	1	15.51688771	3717.152527	28264.32934	39493.12	2062.889	38.7994494
MiCOMT11	1	1.885523736	107.2092505	287.9592723	727.9166	57.0426	16.34123482
MiCOMT12	1	9.266092668	247.7942869	2412.298466	26.34634	14.30462	100.6035238
MiCOMT13	1	19.21769772	2621.872354	23377.20516	217.6127	25.02577	182.4725505
MiCOMT14	1	5.469652114	327.9689707	1395.057141	1.598752	6.740044	345.3609269
MiCOMT15	1	4.111218474	204.8815252	1943.420703	0.702623	7.727253	149.6298353
MiCOMT16	1	3.262322836	1.084858336	27.96081925	0.419053	7.259388	0.039734745
MiCOMT17	1	2.048524741	29.23160714	36.44317954	9207.382	3501.272	166.1135369
MiCOMT18	1	2.174017979	108.0471587	7381.695604	6266.157	1660.702	1211.427772

Table S6. Expression patterns of *MiCOMTs* genes under the treatment of ABA.

Gene name	0h	3h	6h	12h	24h	48h	72h
MiCOMT1	1	0.166997252	0.687179693	0.218312633	4.252705093	9.737157	7.544352095
MiCOMT2	1	0.193099197	0.905331333	0.348427134	22.96842049	1.405399	2.336092774
MiCOMT3	1	0.159942066	0.360647918	0.159982741	9.844237954	8.960678	3.146965105
MiCOMT4	1	0.188452116	0.668824384	1.65876E-05	0.056838275	0.01256	1.077230145
MiCOMT5	1	2.866517704	71.98832701	0.732291231	119.6328377	1.649001	8.244301508
MiCOMT6	1	0.256617778	0.55144956	0.00108743	1.274864874	0.276156	0.393977501
MiCOMT7	1	0.23410366	2.235338342	0.000750061	0.420568662	0.365475	15.89559164
MiCOMT8	1	0.833759204	3.330467646	0.437263401	856.9236855	1.207161	0.250359863
MiCOMT9	1	0.656064083	4.7580214	0.831263685	1555.367798	4.715478	0.520660812
MiCOMT10	1	0.186265509	0.780150348	0.188282772	151.0263384	0.168984	0.679065393
MiCOMT11	1	0.341819965	2.127922709	0.270233892	926.3580758	1.57181	0.274265076
MiCOMT12	1	0.044329818	2.986927533	1.943601707	4161.597955	278.3003	75.67137184
MiCOMT13	1	0.039362609	6.623082471	3.708778641	11416.53078	357.5233	64.48822063
MiCOMT14	1	0.065081196	12.09639695	4.602135842	31290.10566	3740.772	3488.86479
MiCOMT15	1	0.251661726	8.968093252	3.990404462	13351.27867	3289.496	4500.318795
MiCOMT16	1	0.182953684	0.254072072	5.139457686	9.942798973	7.78952	1.230692982
MiCOMT17	1	0.229553387	0.569467593	30.47698074	156225.0219	29786.76	10404.52625
MiCOMT18	1	0.851344691	0.294156799	2.307133898	47404.81545	38704.68	4938.058946

Table S7. Expression patterns of *MiCOMTs* genes under the treatment of SA.

Gene name	0h	3h	6h	12h	24h	48h	72h
MiCOMT1	1	1.566486292	0.054949602	0.289360528	1.369547318	0.699848125	0.057449481
MiCOMT2	1	0.565546614	2.342900432	1.745823885	0.035118221	0.134604643	1032.237795
MiCOMT3	1	1.766762192	0.158802175	0.456500575	0.536558698	0.867709263	0.835448888
MiCOMT4	1	7.502738614	175.0584123	0.085155223	0.100116106	0.096665911	2265.235777
MiCOMT5	1	204.7394588	9745.44992	480.0267448	11.42338193	1.201897167	23272.59518
MiCOMT6	1	52.3835211	1131.304928	29.82414532	12.45502473	11.99329918	69180.50632
MiCOMT7	1	0.037814822	20.54266142	0.074955613	0.009699624	0.043041802	246.0347874
MiCOMT8	1	8.904649449	1.945812806	0.000690553	0.068295551	0.033134291	681.5437718
MiCOMT9	1	5.249958469	23.93908798	0.066762264	0.131021853	0.073421329	751.4269501
MiCOMT10	1	0.36388475	1.861962327	1.825067476	0.003695836	0.000465194	150.4949608
MiCOMT11	1	5.093909472	27.36080838	0.02703224	0.054976489	0.028757267	366.0703772
MiCOMT12	1	0.506607817	46.12801674	0.218851969	0.025450913	0.048065151	240.126469
MiCOMT13	1	1.54516396	18.92513918	0.142101318	0.049110909	0.003043699	14.07087407
MiCOMT14	1	1.420881604	37.54715028	0.1406493	0.002884458	0.003417295	25.9607012
MiCOMT15	1	0.334014376	7.850832871	0.035374869	0.004250294	0.005341926	15.21226221
MiCOMT16	1	0.002810066	2.735923021	0.001830476	0.001230456	0.0000789030	29.76515119
MiCOMT17	1	0.001345805	1.284936901	0.258795108	0.233824346	0.122340646	5359.332937
MiCOMT18	1	0.000318138	0.015197978	0.065090748	0.024175532	0.007234868	368.6096714

Table S8. Expression patterns of *MiCOMTs* genes under the infection of *Xanthomonas campestris* pv. *mangifer-aeindicae* condition.

Gene name	0h	3h	6h	12h	24h	48h	72h
MiCOMT1	1	0.050073034	0.056152991	0.007000536	0.365810308	2.138716	0.007718967
MiCOMT2	1	2.885196057	3.100052031	0.079881691	22.44109926	5.02962	6.684792939
MiCOMT3	1	0.046483852	0.061707091	0.017600636	1.277031974	4.52537	0.5111355
MiCOMT4	1	0.25141187	0.109132363	9.926595508	39617.39248	1.22265	3.65843823
MiCOMT5	1	7.933983376	22.49055218	151.4059174	636330.6281	34.53136	94.66927852
MiCOMT6	1	0.313780606	0.148925298	3.973658869	14749.02624	6.800237	1.968667223
MiCOMT7	1	2.745362997	0.640267495	0.346614615	12219.54567	45.53478	11.1791788
MiCOMT8	1	0.012628996	0.034860055	30.52481894	27374.61387	9313.577	34036.88708
MiCOMT9	1	0.0347363	0.138527125	40.9688137	12478.75718	8161.181	20113.41542
MiCOMT10	1	0.000123982	0.00089736	0.008396412	134.8331954	7.334487	224.1540982
MiCOMT11	1	0.126312946	0.233412327	22.07517433	26516.78459	12915.23	87539.95594
MiCOMT12	1	0.05236811	0.012408393	0.014065278	1811.521191	670.88	425.6694694
MiCOMT13	1	0.754490106	0.053556676	0.128090812	13282.6916	1935.562	3724.664317
MiCOMT14	1	0.049605365	0.004165142	0.115988596	2006.738624	1219.949	1664.824139
MiCOMT15	1	0.043847099	0.004648936	0.143703405	1783.777034	1740.593	1648.134389
MiCOMT16	1	9.872555436	0.117874008	387.483479	112.1619356	360.8281	6677.593041
MiCOMT17	1	0.047960499	0.036276141	0.050874803	818.2597772	20.71377	15.59693577
MiCOMT18	1	503.2268993	0.014768087	0.857101698	32.05792745	2.861829	1.972287465

Table S9. Expression patterns of *MiCOMTs* genes under the infection of *Colletotrichum gloeosporioide* condition.

Gene name	0h	3h	6h	12h	24h	48h	72h
MiCOMT1	1	1.345097001	0.254741959	0.594069243	1.357351144	0.504742	0.223747318
MiCOMT2	1	0.022958871	1735.672963	2.837572216	12.21470188	0.454837	3.037748745
MiCOMT3	1	6.376512727	0.255425205	1.628060425	6.031560066	4.919965	2.268011
MiCOMT4	1	0.971474143	9.118989263	0.259039173	0.415699436	0.527976	0.48461541
MiCOMT5	1	0.157958613	22.60832925	7.169143101	6.463140306	0.86862	0.338815236
MiCOMT6	1	1.619936739	7.628057613	0.58114105	3.63192313	1.145608	1.091697329
MiCOMT7	1	27.95714751	59.44552052	1.227351735	32.99982676	3.384679	6.403794907
MiCOMT8	1	2.062681873	25.20318414	3.912453287	18.27349781	4.756351	0.098516167
MiCOMT9	1	0.42203615	5.692949311	0.152957882	1.236568197	0.528765	0.036768903
MiCOMT10	1	1.52528085	5.083569021	2.311318496	21.99132011	1.238295	0.018010541
MiCOMT11	1	0.571708792	12.29407669	0.214906289	1.322420575	1.326523	0.049087649
MiCOMT12	1	0.245076696	5.498777441	1.178790412	6.94944253	1.642669	3.413478766
MiCOMT13	1	0.292202116	6.15069922	1.278641966	3.115227532	3.759603	4.034282006
MiCOMT14	1	0.759446892	7.926501037	0.172825365	1.86100007	1.405043	4.027244222
MiCOMT15	1	0.932796347	147.5769441	132.7646032	6.771074492	20.62721	22.82565018
MiCOMT16	1	1.253946485	5.14466736	2.17114758	0.899149328	0.03677	1.023482677
MiCOMT17	1	11.98713277	3619.054568	151718.081	84452.26765	28178.86	91083.36226
MiCOMT18	1	0.571711059	8.636295441	7698.903466	1761.675691	336.2108	1263.93104
